# RareLSD: a manually curated database of lysosomal enzymes associated with rare diseases

**DOI:** 10.1093/database/baz112

**Published:** 2019-11-04

**Authors:** Sana Akhter, Harpreet Kaur, Piyush Agrawal, Gajendra P S Raghava

**Affiliations:** 1 Department of Computational Biology, Indraprastha Institute of Information Technology, New Delhi 110020, India; 2 CSIR-Institute of Microbial Technology, Chandigarh 160036, India

## Abstract

RareLSD is a manually curated database of lysosomal enzymes associated with rare diseases that maintains comprehensive information of 63 unique lysosomal enzymes and 93 associated disorders. Each entry provides a complete information on the disorder that includes the name of the disease, organ affected, age of onset, available drug, inheritance pattern, defected enzyme and single nucleotide polymorphism. To facilitate users in designing drugs against these diseases, we predicted and maintained structures of lysosomal enzymes. Our information portal also contains information on biochemical assays against disease-associated enzymes obtained from PubChem. Each lysosomal entry is supported by information that includes disorders, inheritance pattern, drugs, family members, active inhibitors, etc. Eventually, a user-friendly web interface has been developed to facilitate the users in searching and browsing data in RareLSD with a wide range of options. RareLSD is integrated with sequence similarity search tools (e.g. BLAST and Smith-Waterman algorithm) for analysis. It is built on responsive templates that are compatible with most of browsers and screens including smartphones and gadgets (mobile, iPhone, iPad, tablets, etc.).

## Introduction

Due to the low prevalence of certain disorders, as low as ~1:1600 and 1:2000 affected individuals by a certain defect in the USA and Europe, respectively, the nomenclature of “rare disease” is most suggestively used for such maladies (1). Despite a minuscule percentage of people suffering from such chronic congenital illnesses, a considerable proportion of people around the world, belonging to various communities, sects and tribes, have been constantly suffering from them over generations. These disorders, apart from bringing down the quality of life of the proband, severely affect the mental and emotional status of the relatives who constantly manage these patients and live with them through it. An appalling number of such cases increase the genetic burden on the economy, eventually leading to its deterioration. A group of such genetic disorders due to malfunctioning of enzymes within the lysosomes are called as lysosomal storage defects.

Christian De Duve, in 1955, serendipitously discovered small membrane-bound organelles in his lab in Belgium and called them “lysosomes” or “lytic bodies.” These organelles were of the order of size in microns precisely between 0.05 and 0.5 μm. Even though these organelles are small, they play a vital role in maintaining the homeostasis of any cell in a eukaryotic organism apart from signaling and secretion (2, 3). The lysosomes get this property from the vast plethora of hydrolytic enzymes they boast of. The proteins within the lysosomal compartment belong to various families such as hydrolases, ceramidases, acid phosphatases, sulfatases, etc., which need an optimum temperature and an acidic environment to vitally functioning inside the cell. The single glycocalyx membrane contains acidic enzymes and separates the lysosomal contents from the rest of the cellular environment (3). Belonging to different families, these enzymes follow different catalytic mechanisms and breakdown specific substrates within the body (3). Any malfunctioning in these vital players eventually results in an accumulation of undegraded substrates in various tissues of the body as inclusions and thereby manifests as lysosomal storage disorders (4). These rare monogenic disorders have prevalence of 1 in 8000 live births globally and are also the second most common group of an inborn error of metabolism with an incidence of as high as 1 in 1500, which makes it a commonly occurring genetic defect (5).

These disorders affect various tissues and systems in the body, with symptoms manifesting in the first decade of life, which eventually worsen over time. Majority of them follow an autosomal recessive inheritance pattern except Fabry’s and Hunter’s (MPS II), which follow an X-linked recessive pattern, whereas Danon disease follows an X-linked dominant pattern of inheritance (6). For an autosomal disorder to manifest in a child, both parents are potential carriers with similar mutations in the gene affecting the functioning of an enzyme in the lysosome. In different parts of the world, particularly in patches in the Middle East and some countries in South Asia such as India and Pakistan, the rate of inbreeding is high particularly due to consanguineous marriage. This results in the transfer of deleterious mutations to subsequent generations over a lineage. Therefore, there is a massive increase of genetic overload particularly due to these lysosomal defects.

**Figure 1:**
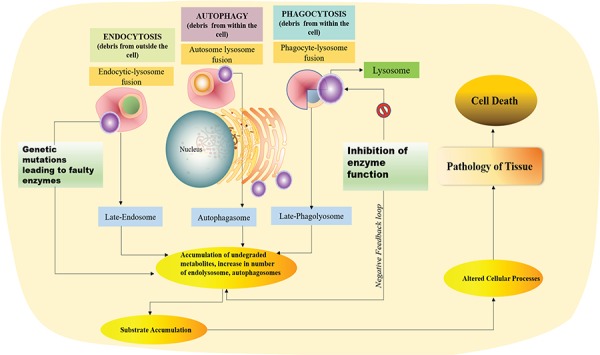
Graphical presentation of debris degradation by lysosomal enzymes and associated disorders due to defect in the lysosomal enzyme.

The signs and symptoms include coarse facies, hypotonia, inability to thrive, poor feeding capabilities, hepatosplenomegaly (elevated liver function tests), intellectual disability, contractures, skin abnormalities (in some disorders), corneal clouding, macular cherry red spot, dysostosis multiplex and many other allied symptomatic manifestations that practically render the proband dependent for the rest of their lives (Figure 1). These symptoms develop mainly due to the accumulation of metabolites, namely, heparan sulphate, dermatan sulphate, keratan sulphate, lipids, proteins, lyso-Gb3, etc. (6). A substantial boom has been witnessed in this bustling area of the genetic defect research, which has led to therapies being actively designed for the same to alleviate the individuals from this misery. Very few drugs for some disorders such as Fabrazyme for Fabry’s, Cerezyme for Gaucher type 1, Aldurazyme for Hurler syndrome (MPS I), etc. (7) are available in the market that have been acting as potential candidates to suppress the distress caused, either symptomatically or helping in prolonging the lifespan of such individuals. Understanding the underlying molecular mechanism responsible for the development of LSD’s would be crucial for the development of efficient new drugs. Hence, it is important to provide researchers a dedicated resource that accumulates scattered information regarding these players, i.e. enzymes associated with LSDs. Till date, there has been no repository that caters entirely to these lysosomal defects from the perspective of the proteins, or otherwise, in spite of their high incidence in various parts around the globe. RareLSD is, therefore, a contribution to the scientific community who has been actively researching the area of lysosomal storage disorders. This is a comprehensive information system that serves as a common portal to address and understand these disorders from the perspective of the enzymes present in these lytic bodies. It encompasses all relevant information about the enzymes from all significant data repositories such as PubMed (8), OMIM (9), BRENDA (2019 update) (10), UniProt (11), MalaCards (version 4.10.0.7) (12) and GeneCards (version 4.10.0 Build 10), (13) along with finely extracted information from the patient case reports that were freely available on the web, all in a single platform. This resource integrates various web-based tools for searching and browsing facilities to facilitate users in extracting and analyzing data. We hope that this portal will be a useful contribution to divert more attention to this field of research and will serve as a one-stop compendium for all the information related to these biocatalysts.

## Material and Methods

### Data collection

The data were manually curated, covering all aspects of the enzymes from four broad perspectives, namely, chemical assays, families, lysosomal storage defect categories and a disorder entity, and were further subclassified on the basis of their inheritance patterns, the organs affected and the age of onset of disorders. PubMed was queried to obtain reviews, articles and hospital case reports to obtain classical information using the keywords “lysosomal storage disorders [Title/Abstract]” and “lysosomal enzymes [Title/Abstract]” and “rare genetic defects LSD [Title/Abstract])” AND “inborn errors of metabolism [Title/Abstract]” AND “enzyme therapy for storage disorders [Title/Abstract]”. It resulted in nearly 20,050 publications; after sorting the queries further based on relevant keywords and identifying recent publications from the last 15 years, we were able to narrow down information from 1,500 articles published until April 2018. We have excluded papers that did not directly cause the disorders from the 63 unique enzymes being looked at *in toto*, to avoid any confusion in understanding.

Information about drugs available against these enzymes in the market or under clinical trials was obtained and incorporated in our database from Orphanet (version 5.24.0) (14) and DrugBank (version 5.0) (15). The variants and single nucleotide polymorphism (SNP) were mined from the articles through PubMed (9), as well as GeneCards (version 4.10.0 Build 10) (13), MalaCards (version 4.10.0.7) (12) and dbSNP (16).

The facts on prevalence pertaining to a specific disorder, the enzyme residual volumes in affected patients, the catalytic residues, modifications present in the protein, the pseudogenes present, existing paralogs and bioassays had been mined manually via various big repositories such as MEROPS (Release 12.1) (17), DisGenET (version 5.0) (18), MalaCards (version 4.10.0.7) (12), PubChem (19), PubMed (8), etc. Tertiary structures of lysosomal enzymes were extracted from Protein Data Bank (PDB) that contain experimentally determined structure of proteins (20). We got structure of most of lysosomal enzymes in PDB with resolution better than 4 Å. The eight enzymes for which structure wasn’t available were predicted using the freely available online software Phyre2 (version 2.0) (21). Secondary structure information of these enzymes was assigned using DSSP software (22, 23).

## Architecture and Interface

**Figure 2:**
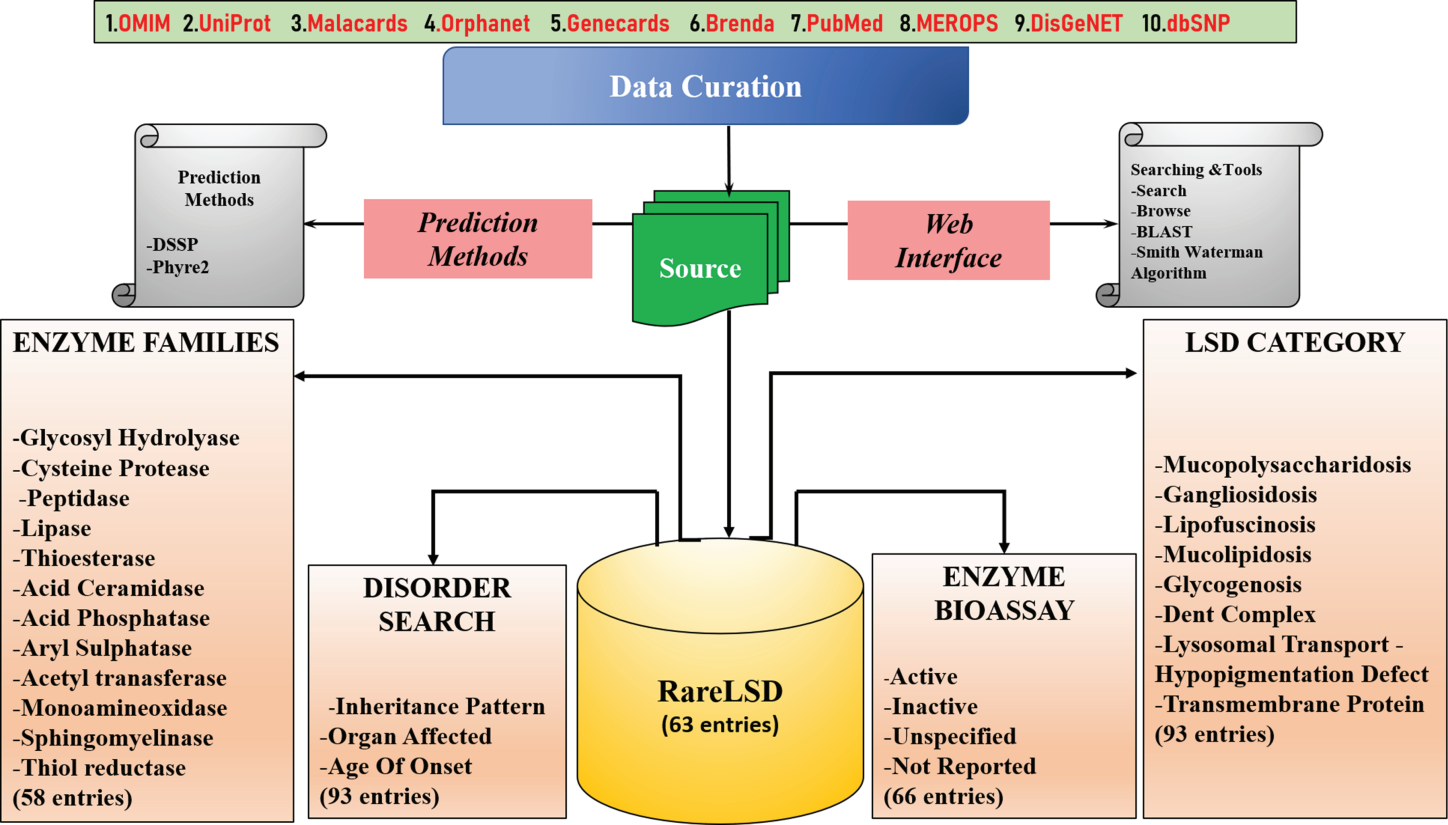
The organization of the RareLSD.

RareLSD was built using Apache HTTP server on Linux Platform. The most popularly available open-source relational database management system (RDBMS), MySQL version 5.7.24, was used at the back-end to organize all data into four tables. The tabular organization of data enabled better and efficient data retrieval and, therefore, clear visualization on the interface. The main table with 33 different fields was maintained associated with each of the 63 unique entries. Four other tables curated in MySQL held information about disorders, the chemical bioassays, age of onset and the last for structural insights about the enzymes. The front-end was maintained using CSS3, HTML5, PHP version 4.0.10 and JavaScript (1.7). Overall organization of the database represented in Figure 2.

The consistency of the interface allows the user to get accustomed to all facilities and tools provided in the database for their informational needs.

## Organization of the database

The data organized in RareLSD falls into four broad categories, namely, LSD category, families, disorder and bioassay:

**I. Lysosomal Storage Defect (LSD Category):** All relevant information about 93 disorders caused by malfunctioning of any of the lysosomal protein are made available, distinguished categorically into nine major categories of lysosomal storage defects such as “mucolipidosis,” “gangliosidosis,” “lipofuscinosis,” “mucopolysaccharidosis,” “glycogenosis,” “dent complex,” “lysosomal transport disorder,” “hypopigmentation defect” and “transmembrane protein.”

**II. Family:** The visualization of enzymatic data based on families of proteins to which they belong to are sorted into 12 protein families, namely, “glycoside hydrolase,” “cysteine proteases,” “peptidases,” “lipase,” “thioesterases,” “acid ceramidase,” “acid phosphatases,” “aryl sulfatases,” “acetyltransferases,” “monoamine oxidase,” “sphingomyelinase” and “thiol reductase” for ease of the user to filter enzymes based on specific families and study their properties.

**III. Disorder:** The disorders, as a whole, can be viewed by the user on the interface based on different categories as following:

**A. Inheritance Pattern:** This page provides the option to browse the enzymes of LSDs based on the inheritance pattern followed by the disorder caused by the malfunctioning or absence of these enzymes like autosomal recessive, autosomal dominant, X-linked recessive, X-linked dominant, Y-Linked and ones that are not inherited.

**Figure 3:**
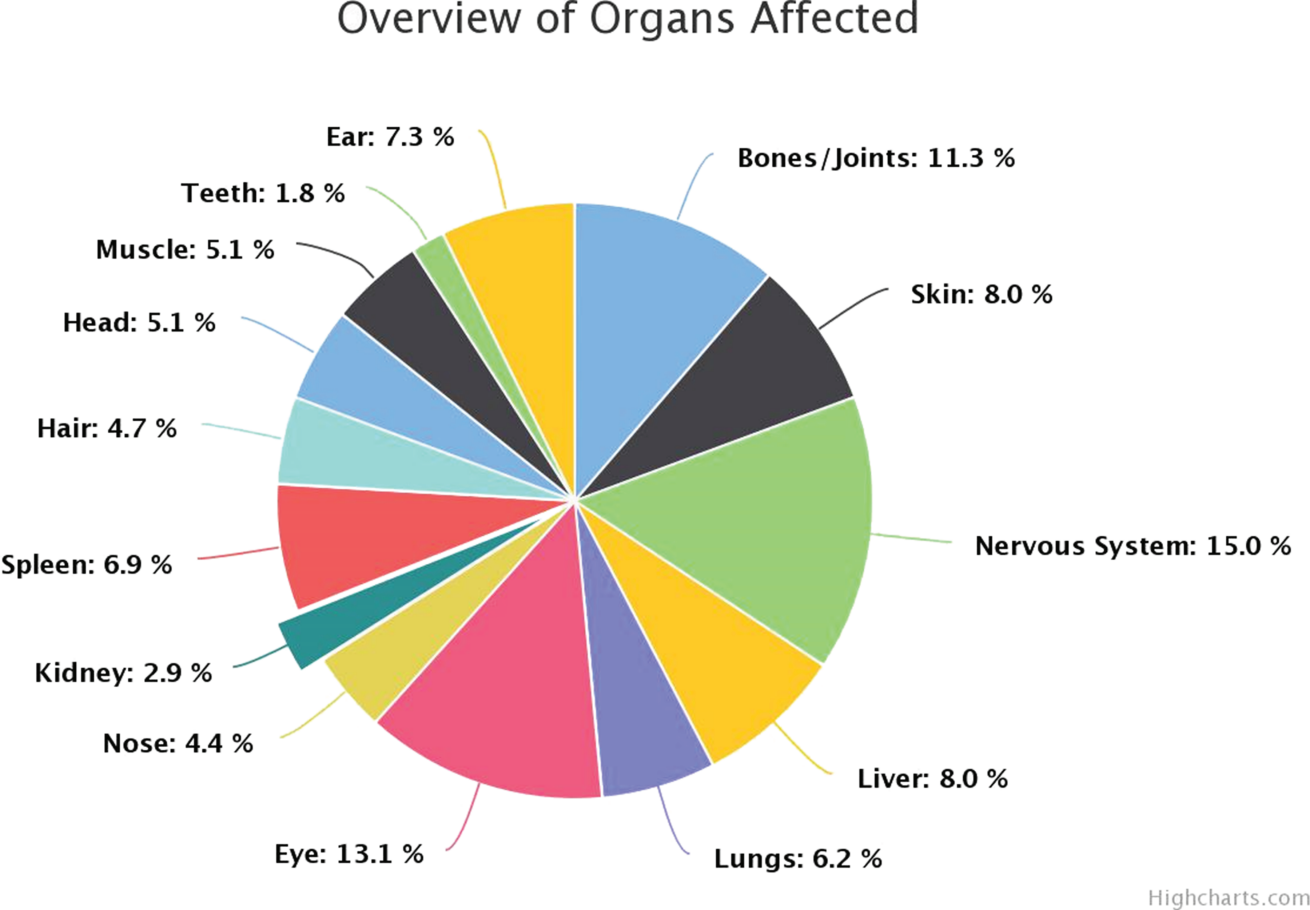
Overview of organ involvement in lysosomal defects.

**B. Organs Affected:** The browsing can be carried out by clicking through an option of 15 affected parts of the body and disorders that are associated with the same as shown in Figure 3.

**C. Age of Onset:** This section allows the user to easily look for “early,” “adult” and “early and adult (both)” onset disorders, at a glance. The section also keeps up with the genotype-phenotype correlation to give a broader perspective of how the mutations in these critical players affect the functionality of vital proteins, which are thereby responsible for bringing down the metabolism of the body.

**IV. Bioassay:** The information in the bioassay section was curated from PubChem. From the total entries retrieved upon searching for a particular enzyme, the “confirmatory” assays were filtered and incorporated as a part of our knowledge pool. Out of the 63 enzymes, information about 49 enzymes was found to exist in PubChem; however, the remaining 14 enzymes did not appear in our search. The assays can be navigated by the user via enzyme name and the number of assays highlighted against each entry. The assay card of each enzyme displays detailed information related to all existing assays against each enzyme.

Under a separate subheading “Publications,” the research articles used to extract the information from have been enlisted in a tabular fashion with hyperlinks to each of their PubMed ID’s, which further redirects these articles to their relevant site domains. Another subcategory maintained under the General section termed as “case reports” can be efficiently used to specifically look for hospital-based case reports, consisting of specific pedigrees, relevant phenotypic images of the disorder, the mutations reported and the plan of action to manage the defect. Each enzyme, the associated disorder and its structure are individually linked to their UniProt, OMIM and PDB IDs wherever available to enable easy and oriented search via the interface.

## Implementation of web tools

Various tools for searching, browsing and analysis were integrated in RareLSD for user friendly experience and hassle-free data searching.

**A. Search tools.**

We have implemented three different modules, namely, “simple search,” “advance search” and “pattern mapping” under the search category:

**A.1. Simple search:** This tool represents keywords for data retrieval module. This can be executed by search coins such as enzyme name, gene, family, drug, disease, metabolite, temperature, cytogenetics, sequence length, PDB ID, etc. Moreover, this module also allows users to select various fields to be displayed for the result.

**A.2. Advance search:** For performing complex searchers using two or more query keyword related to the enzyme, the user can perform multiple structured query system using the advanced search option of our web portal. It performs four query searches simultaneously, by default, but it also allows the user to select a desired field/keyword against which search can be established. Apart from this facility in the advanced search module, it allows the user to apply basic standard logical operators (e.g. like, >, < and =). Additionally, the queries to be implemented can also be added or removed to perform precise searches.

**A.3. Pattern mapping:** This is a platform for searching a given peptide sequence against all enzyme sequences available in RareLSD. It searches for a pattern matches of given peptide sequences against enzyme sequences in the database. The hits retrieve those enzymes sequences that contain the query peptide.

**B. Browse tools:** We have implemented a browsing facility that helps the user for convenient data navigation within the database in an orderly manner. In this module, a user can retrieve information on lysosomal enzymes by browsing four main categories: (i) lysosomal storage disorder category, (ii) family, (iii) disorder and (iv) bioassay.

The “bioassay” field allows the user to retrieve all relevant information about the all existing assays conducted on the lysosomal enzyme, the gene encoding it, the total number of “confirmatory assays” present and their activity status. Based on which metabolite gets accumulated and leads to enzyme dysfunction, LSD’s have been categorized. It also tells the presence of mannose-6-phosphate residues if available for each protein. The “family” field offers the user to fetch the information on the family to which the enzyme belongs to phylogenetically along with their cytogenetic location. Also, lastly, the disorders have been summed up critically in the “disorder” category, which are further searched on the basis of (a) inheritance pattern, (b) organs affected and the (c) age of onset.

**C. Similarity:** This module facilitates the user to perform various analyses, such as sequence similarity, by implementing different web-based tools, i.e. Basic Local Alignment Search Tool (BLAST) and Smith-Waterman algorithm in RareLSD database.

**C.1. BLAST Search:** Using different parameters such as weight matrix and an expectation value, the BLAST search can be executed in our database, with peptides submitted in the FASTA format (24). This tool offers a user to execute a similarity-based search against RareLSD database.

**C.2. Smith-Waterman Search:** This algorithm executes a similarity search against small peptides more efficiently using the Smith-Waterman algorithm. This module permits the user to search peptides in our database similar to their query peptides. In this option, a user can submit simultaneously multiple peptide sequences in FASTA format (25).

## Database statistics

RareLSD is composed of a total of 63 unique enzyme entries, which can be found in the human lysosomal environment. However, compiled data represents a total of 67 enzyme entries, since two enzymes play role in other diseases (63 + 4) each causing a unique genetic disorder. Further, the maximum length of the amino acid sequences out of the curated enzymes was 1256 amino acid residues for *N*-acetylglucosamine-1-phosphotransferase subunits alpha/beta (E.C. number 2.8.7.17), encoded by the gene GNPTAB. The smallest protein sequence of 193 residues, on the other hand, was of ganglioside GM2 activator (E.C. number 3.2.1.52), encoded by gene *GM2A.*

Certain amino acid residues in a protein play a significant role in lots of aspects related to either protein maturation, imparting catalytic activity, signalling, trafficking, etc. (26). For instance, two residues, namely, methionine and cysteine, have been assessed in the proteins within the lysosomal niche. The methionine at the start of each protein sequence is cleaved by enzyme methionine aminopeptidase (MAP) (23). The thiol group of cysteine residues (Cys) participates in the formation of disulfide bridges that maintain the quaternary structure of enzyme, leading to its active nature, as the information regarding cysteine is available only for 49 enzymes in the literature. Thus, we have maintained these data for these enzymes only in our database. Apart from a proper structure, the functionality of these biocatalysts is highly regulated under optimum temperature and pH conditions. The average temperature for optimal functioning was found to be 37°C within this double membrane-bound organelle, which had an acidic pH of around 5.5.

The structures reported in our database for 55 enzymes were extracted directly from RCSB PDB portal, whereas the remaining eight enzymes, for which the structures were not available, were predicted via a web-based software called Phyre2, with an average confidence and coverage score of 92.97% and 75.14%, respectively. The compilation of the reported secondary structure content percentage in RareLSD against each structure computed either by PDB or Phyre2 was made using the DSSP program (20–22). The average helix, sheet and coil content across all the 63 entries were found to be 30.32%, 23.30% and 46.38%, respectively. This is indicative of the fact that coils dominate the secondary structure amongst these biocatalysts inside the lysosomal proteome.

**TABLE 1:** Prevalence of most common lysosomal defects

**S. No**	**Enzyme**	**Gene**	**Disorder**	**Prevalence**
1	Alpha galactosidase A	*GLA*	FABRY	1–5/10000
2	Iduronate 2 sulphatase	*IDS*	MPS2	1–5/10000
3	Cathepsin D	*CTSD*	NCL	1/12500
4	Acid alpha-glucosidase	*GAA*	POMPE	1/40000
5	Sphingomyelinase	*SMPD1*	NEIMANN PICK A	1/2 50 000
6	Sphingomyelinase	*SMPD1*	NEIMANN PICK B	1/2 50 000
7	*N*-acetylsphingosine amidohydrolase (acid ceramidase) 1	*ASAH1*	FARBER	<1/1000000
8	Beta-galactosidase	*GLB1*	GM1 type 2	<1/1000000
9	Glucosylceramidase	*GBA*	GAUCHER	1–9/100000
10	Alpha l-iduronidase	*IDUA*	MPS1	1–9/1000000

Post-translational modifications in proteins are an important aspect of enzyme maturation, leading to an active enzyme (27). The reported enzymes in RareLSD have found to undergo heavy glycosylation across all entries, with asparagine (Asn) being the most common residue to undergo N-linked glycosylation in each entry, except Sialidase 4 (E.C. number 3.2.1.18), encoded by gene *NEU4*. Other modifications such as ubiquitination and phosphorylation have also been frequently reported in the literature.

The highly heterogeneous protein pool of the lysosome in terms of its functionality is composed of 51% of the proteins as catabolic enzymes, the remaining proportion is taken up by proteins in the transmembrane region as transport proteins and as accessory proteins. Defects in any of these proteins are known to disturb the hemostasis, leading to disorders of storage and metabolism. The manifestation of defects caused by the malfunctioning of these enzymes is categorized largely from literature-curated data into eight broad categories based on the accumulated metabolite. The most frequently occurring defect was found to be mucolipidosis ~30%, followed by lipofuscinosis reported in 12% disorders, and hypopigmentation and defects caused by transmembrane proteins in the lysosome accounting for 5% of each category of the disorders. On an average, 48% of all proteins in the lysosomal environment have the presence of a mannose-6-phosphate residue required for proper trafficking in and out of the lysosome and functioning of the molecules. The amalgamated data about the organ and organ systems affected due to enzyme dysfunction reflect that, on an average, the nervous system is the most affected in these defects corresponding to 15% of all the disorders curated. A peculiar cherry red spot was also observed as an associated symptom along with corneal clouding in 11% of all rare lysosomal defects. This coherently falls in lieu with the literature findings. Apart from the involvement of eye and the nervous system, bones, spleen liver and the skin were found to be frequently compromised in such syndromes. The inferences about the inheritance pattern of these defects follow the typical trend wherein in most disorders, and in our curated instances, 84.5% of known disorders follow autosomal recessive inheritance patterns. A fairly considerable percentage ~12% of disorders follow autosomal dominant patterns, for example, keratolytic winter erythema, narcolepsy 1, Charcot-Marie-Tooth disease 2V (CMT2V), cercarial dermatitis, etc., but these disorders are mostly caused by the non-enzymatic pool of the lysosome. In our disorder pool, three disorders, namely, Fabry’s, Hunter’s, NCL (Kuf disease; adult variant) and Danon disease, follow X-linked inheritance as an exception to the general trend. The distinct prevalence of disorders globally can be looked on a world map under the title of “prevalence map” on the website. The top 10 enzymes associated with the most prevalent disorders were tabulated in Table 1.

## Availability

The website is responsive and compatible with all the latest gadgets. All prospective users of the data provided in the database such as students, researchers, clinicians, genetic counselors, etc. can access the interface without any *a priori* login credentials required. RareLSD can thus be accessed freely at https://webs.iiitd.edu.in/raghava/rarelsd/.

## Discussion

**Figure 4:**
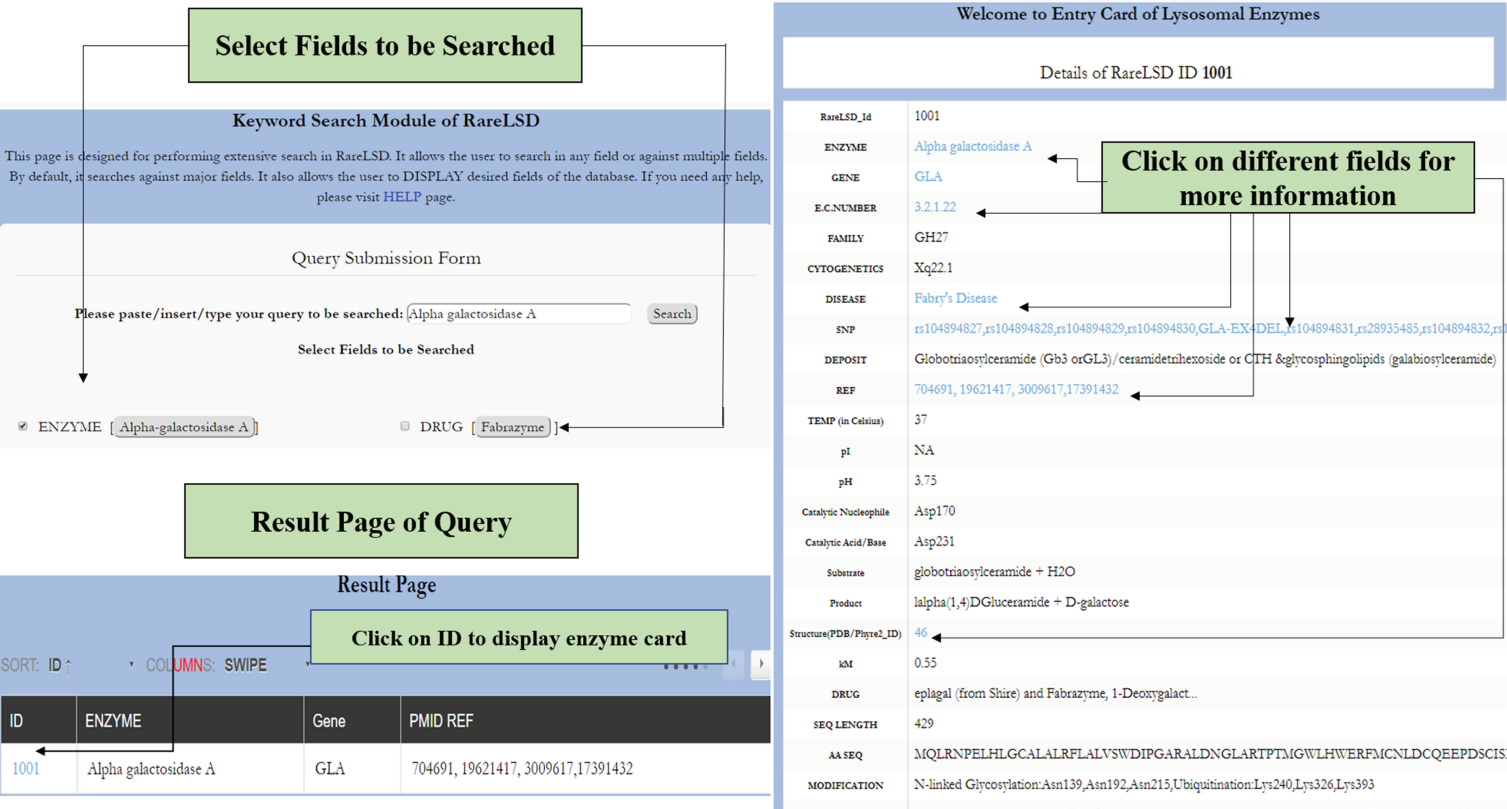
The workflow for simple search in RareLSD.

Working on rare diseases is an upcoming arena in the field of genetics. Any alteration in the genetic makeup of the enzyme-encoding lysosomal gene leads to eventually hampering the cellular hemostasis. This could be extremely fatal during the course of its manifestation. Detecting novel mutations and developing inexpensive and relevant therapies from the existing knowledge pool are necessary to alleviate the burgeoning population by current genetic burden from such enzymes. Thus, lysosomal enzymes need rigorous research such that forthcoming obstacles while developing management therapies are looked at carefully. This platform provides a collection of relevant information that spans all possible aspects of this small niche of biocatalysts housed in the lysosomes. It stores information from the prevalence of widely occurring disorders and a resource tool referred to as Pattern mapping that shall enable hunt for searching peptide matches, which could further be exploited for developing chaperone, ligand-based and enzyme therapies. Other tools, such as BLAST and Smith-Waterman algorithm, enable the user to perform similarity search against the sequences present in the database. Primarily, it shall steer the avenue of research towards devising upcoming therapies for management purposes. The database spans all 63 enzymes; defects causeda by them were explored through various angles. It shall also serve as a vitala resource to mine coherent information. Furthermore, particular protein analysis with its secondary structure content, their active site residues, information about the presence of inhibitors and other aspects of the enzyme, etc. makes it a one-stop compendium for researchers, geneticists, clinicians and counselors rigorously working in this research niche. For instance, Figure 4 depicts how the “simple search” modality for an enzyme, such as alpha-galactosidase A, would work in RareLSD. The display card for the same shows all the information about the enzyme at a glance in a tabular format.

**Figure 5:**
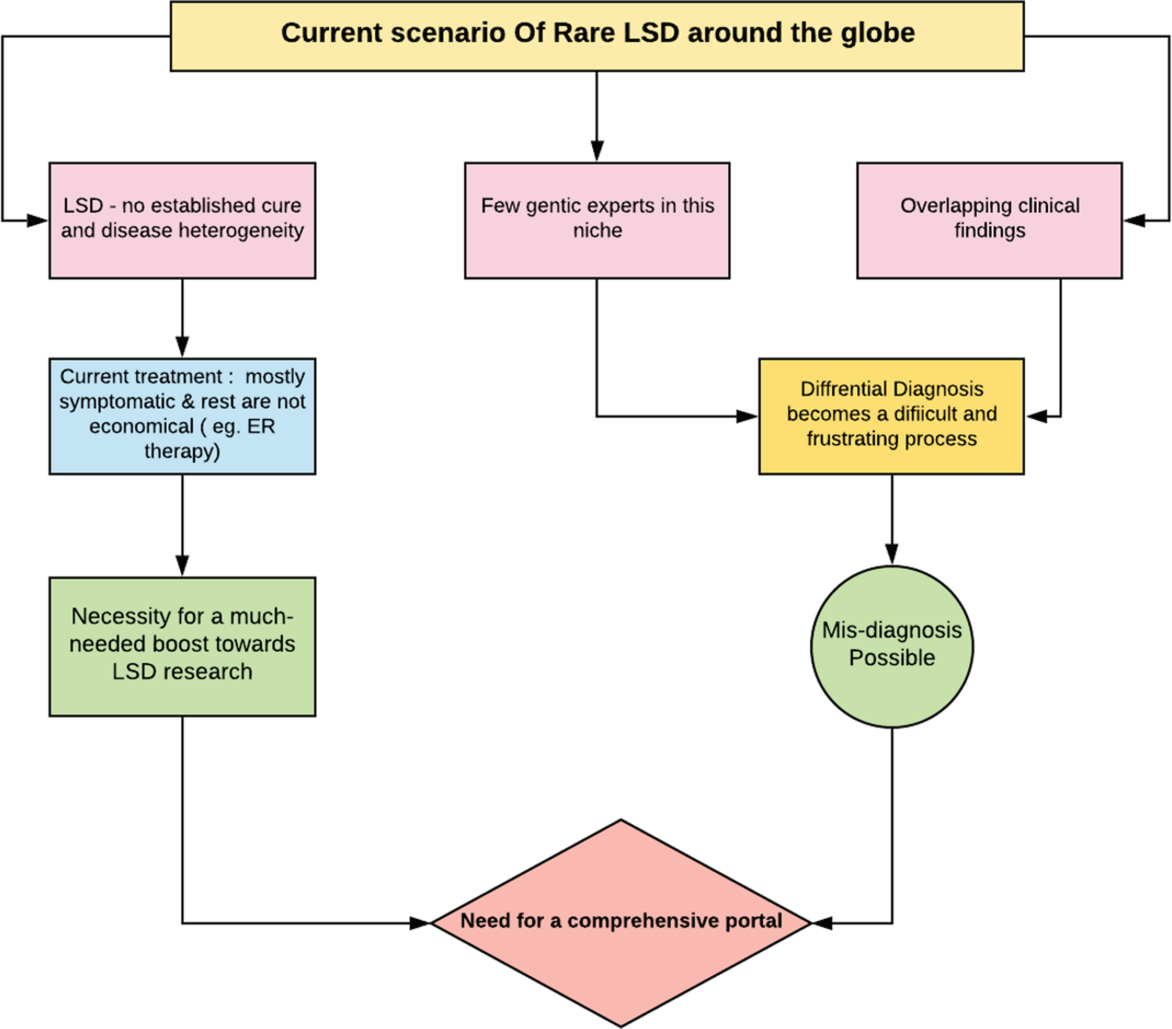
Need for single web portal, RareLSD.

## Utility of database

Potential applications of RareLSD are as following:

1. To the best of the author’s knowledge, currently no database exists that cater solely to the needs of niche research groups working actively in the domain of inborn errors of metabolism specifically lysosomal defects.
2. The tabular visualization of all disorders from the perspectives of causative enzyme allows the researcher or clinician to connect all the dots using this platform.
3. A data portal with a compendium of all information about the lysosomal catalysts at a single stop would make it hassle free and save time to mine the vast information pool dissipated on the internet and draw further inferences about them.
4. Since these disorders manifest at an early age, we need more genetic experts and greater awareness; this portal is thus a small contribution that would divert the attention of clinical practitioners and existing genetic experts to divulge deeper inferences in this domain using our platform.
5. Our web portal provides an opportunity to look for therapeutic peptides across 63 unique gene sequences responsible for causing a certain defect due to lysosomal enzyme dysfunction.

## Case Study

In the current scenario, where the field of rare lysosomal disorders is now slowly grabbing attention, there is an immediate need for a web portal that would change the discourse of attending to these diseases and learning about them. In the absence of RareLSD, a researcher would have to visit different websites at a given time point to make inferences and read about different parameters regarding the enzyme. The inundating information available by most web services is in the form of bulky texts, which take time to comprehend. The tabular display of information on our website makes it convenient to glance at all important features of the lysosomal protein in hand clearly and in a hassle-free manner.

**Figure 6:**
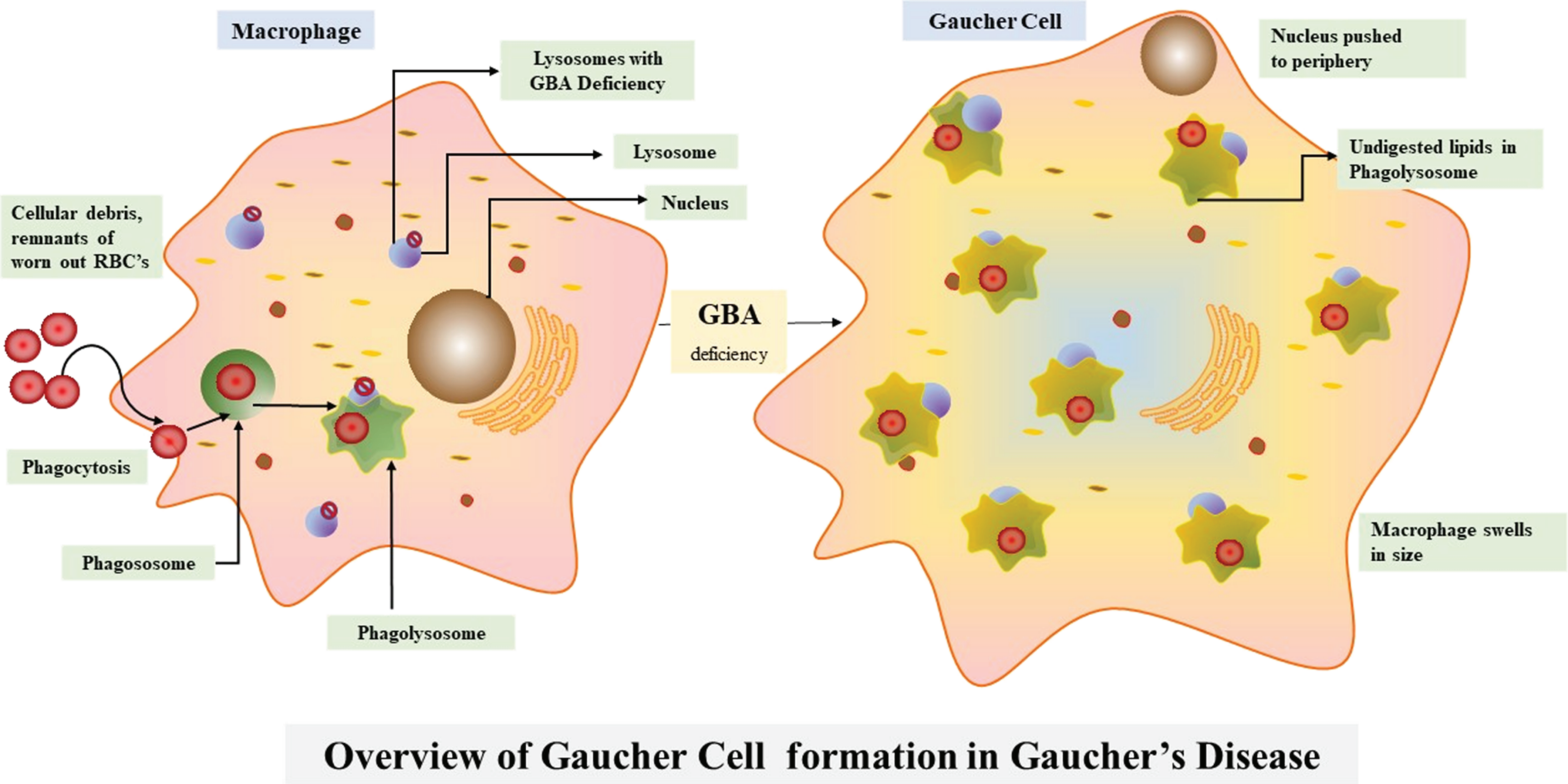
Overview of the Gaucher disease pathway.

Figure 5 broadly suggests the necessity for a comprehensive web portal that would give a much-needed boost to the niche of researchers working with lysosomal defects. RareLSD uncomplicates the exercise of collection and retrieval. The overwhelming amount of data spread across the internet in various repositories is dispersed. RareLSD makes it convenient for the user, as it gathers and displays the information from all these repositories at once, under a single umbrella. This shall save time and make the process of information retrieval via scattered literatures and weblinks less frustrating. For instance, while learning about Gaucher disease, which is caused by the malfunctioning of glucosylceramidase and manifests in early childhood, one can look up our portal for the enzyme glucosylceramidase, the associated gene *GBA* that undergoes various types of mutations like rs104886460, rs1057519020, etc. Any mutation in the GBA gene causes malfunctioning of the proteinaceous enzyme leading to its complete absence or reduced expression (28). Furthermore, various aspects of the enzymes, like the assays, optimum temperature, pH, pI, the catalytic residues, disulfide bridges, structure, etc. can be mined by the user. In a healthy person, this enzyme causes hydrolysis of glucocerebroside that makes up the RBC membranes. Due to the absence of the enzyme, glucocerebroside gets accumulated in the macrophages present in liver, spleen, bone marrow, kidney, etc., ultimately bringing down the quality of the life of the proband. Overview of the pathway associated with Gaucher disease is represented in Figure 6.

**Figure 7:**
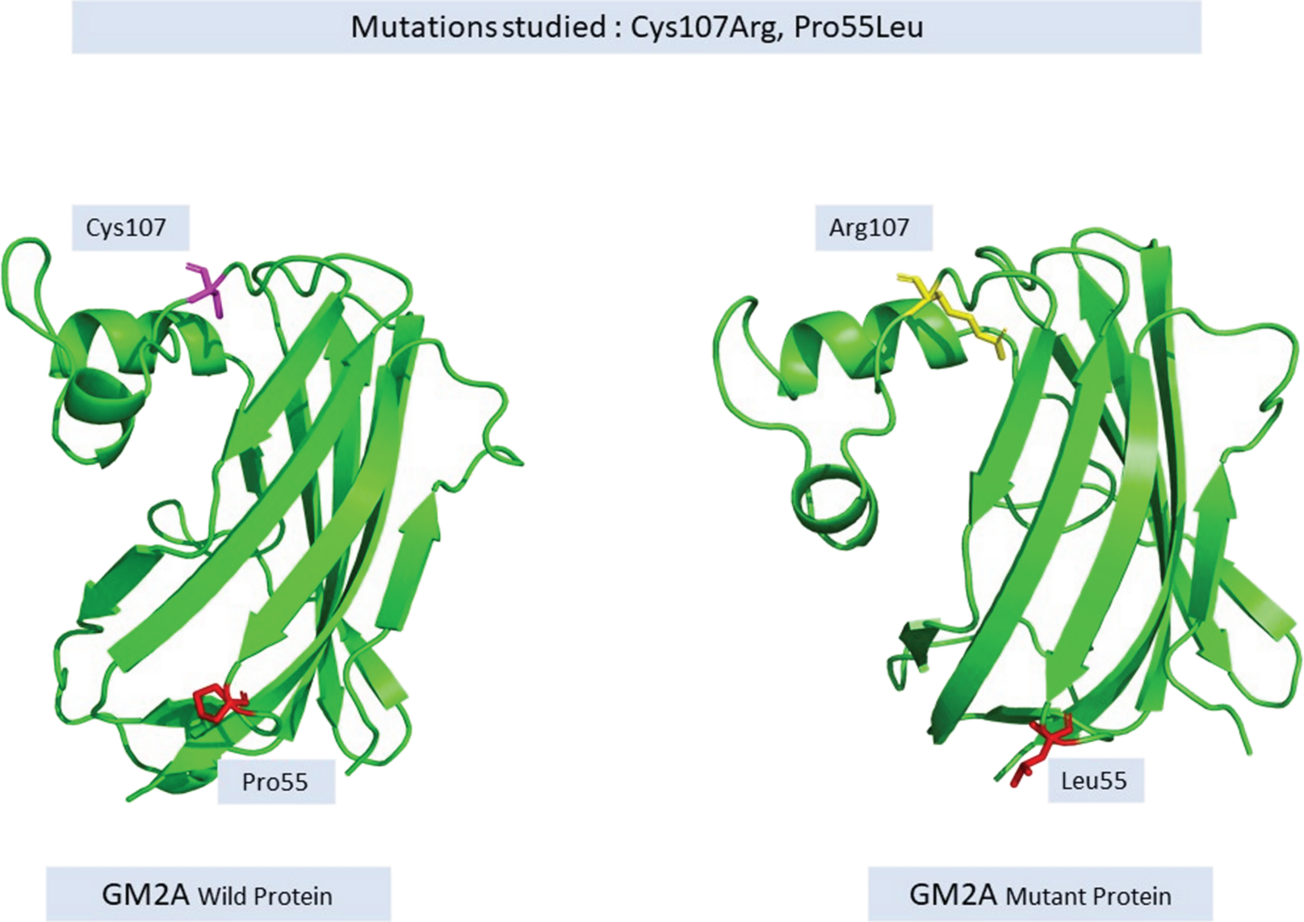
Change in the structure of wild versus mutant GM2A protein.

Changes in a single amino acid also causes perturbations in the protein structure thereby affecting its function, for instance, a protein-like GM2A responsible for causing Gm2-gangliosidosis, Ab Variant, is an autosomal recessive disorder. Most commonly occurring changes, namely, Cys107Arg (TGT- > CGT) and Pro55Leu (CCT- > CTT), lead to disruption in disulfide bridge formation and reduces the interaction of the activator protein to HEXA (29) respectively as shown in Figure 7.

### Update of RareLSD

RareLSD will be continuously updated with sufficient literature accumulation. This will ensure high level and updated quality of information from current research trends in the area of lysosomal genetic defects.

## Contributions

S.A. manually collected and curated all the data. S.A. and H.K. analyzed the data. S.A. and P.A. predicted and analyzed the structures. S.A. and H.K. developed the web interface. S.A., H.K. and G.P.S.R. prepared the manuscript. G.P.S.R. conceived the idea, planned and coordinated the entire project.
